# Cardiac magnetic resonance feature tracking global and segmental strain in acute and chronic ST-elevation myocardial infarction

**DOI:** 10.1038/s41598-022-26968-4

**Published:** 2022-12-31

**Authors:** Jennifer Erley, Jitka Starekova, Martin Sinn, Kai Muellerleile, Hang Chen, Phillip Harms, Lieda Naimi, Mathias Meyer, Ersin Cavus, Jan Schneider, Stefan Blankenberg, Gunnar K. Lund, Gerhard Adam, Enver Tahir

**Affiliations:** 1 Department of Diagnostic and Interventional Radiology and Nuclear Medicine, University Medical Center Hamburg-Eppendorf, Martinistraße 52, 20251 Hamburg, Germany; 2 Department of Radiology, University of Wisconsin-Madison, Madison, WI USA; 3 Department of Cardiology, University Medical Center Hamburg-Eppendorf, Hamburg, Germany

**Keywords:** Cardiology, Diagnostic markers, Translational research

## Abstract

Strain is an important imaging parameter to determine myocardial deformation. This study sought to 1) assess changes in left ventricular strain and ejection fraction (LVEF) from acute to chronic ST-elevation myocardial infarction (STEMI) and 2) analyze strain as a predictor of late gadolinium enhancement (LGE). 32 patients with STEMI and 18 controls prospectively underwent cardiac magnetic resonance imaging. Patients were scanned 8 $\pm$ 5 days and six months after infarction (± 1.4 months). Feature tracking was performed and LVEF was calculated. LGE was determined visually and quantitatively on short-axis images and myocardial segments were grouped according to the LGE pattern (negative, non-transmural and transmural). Global strain was impaired in patients compared to controls, but improved within six months after STEMI (longitudinal strain from −14 ± 4 to −16 ± 4%, *p* < 0.001; radial strain from 38 ± 11 to 42 ± 13%, *p* = 0.006; circumferential strain from −15 ± 4 to −16 ± 4%, *p* = 0.023). Patients with microvascular obstruction showed especially attenuated strain results. Regional strain persisted impaired in LGE-positive segments. Circumferential strain could best distinguish between LGE-negative and -positive segments (AUC 0.73- 0.77). Strain improves within six months after STEMI, but remains impaired in LGE-positive segments. Strain may serve as an imaging biomarker to analyze myocardial viability. Especially circumferential strain could predict LGE.

## Introduction

Myocardial infarction (MI) remains the most common cause of heart failure, despite improvements in acute treatment strategies^1^. Cardiac magnetic resonance imaging (CMR) is a non-invasive modality to visualize the area of damaged and viable myocardium after MI, useful to follow-up on patients after acute MI treatment (for example with percutaneous coronary intervention). Furthermore, CMR is used to detect possible complications and long-term effects of MI on cardiac function^2^.

An established parameter of myocardial function is the left ventricular ejection fraction (LVEF)^3^. Myocardial strain is a novel functional parameter and quantifies the shortening or thickening of heart-muscle fibers from end-diastole (ES) towards end-systole (ES), expressed in per cent change^4^. Due to the different orientations of myocardial fibers, longitudinal, radial, and circumferential strain are determined separately^5^. In comparison to LVEF, strain enables the detection of subtle changes in myocardial function and more so of regional changes^6^. It can be determined by CMR and echocardiography on a global and segmental basis, using various techniques. While most CMR-based techniques require the acquisition of additional pulse sequences, feature tracking (FT) allows strain analysis to be performed on routinely acquired cine series ^7^.

Late gadolinium enhancement (LGE) is another CMR-specific tool, employed to differentiate between viable and irreversibly damaged myocardium following MI, depending on the accumulation of a gadolinium-based contrast agent in infarcted tissue^8^. Currently, myocardial segments with $\le$ 50% LGE extent are classified as viable^9^. Changes in myocardial strain from the acute to the chronic stage of MI, as determined by FT CMR, have not been sufficiently studied. Furthermore, the utility of strain in predicting LGE at different stages of MI remains to be elucidated. The objectives of this study were to 1) determine changes of global/ segmental strain and LVEF in patients after six months following acute ST-elevation MI (STEMI) compared to healthy controls; and 2) analyze the ability of strain at baseline and follow-up to discriminate between LGE-negative and -positive myocardial segments.

## Materials and methods

### Patient population

Patients with initial STEMI were prospectively enrolled from April 2011 to June 2015 (n = 33). The population has been analyzed previously in a study published by *Tahir *et al*.*^10^. Healthy volunteers were enrolled from October 2010 to December 2016 to provide an age- and gender-matched control group. All subjects gave written informed consent. The study was approved by the local ethics committee of the medical association Hamburg and complied with the Declaration of Helsinki. The study was performed in accordance with all relevant guidelines and regulations.

### CMR imaging

Patients and controls who did not meet the exclusion criteria (no informed consent, participation in a pharmaceutical study, hemodynamically unstable at baseline, severe obesity, claustrophobia and presence of a pacemaker and intracranial metal) were invited to participate in the study. Patients received a baseline CMR exam at 8 ± 5 days after the first-time, reperfused STEMI and a follow-up study after 6 ± 1.4 months. The control group received one CMR exam. All subjects were scanned using a 1.5 Tesla scanner (Achieva; Philips Medical Systems). The acquisition protocol included a standard steady-state free-precession (cine-SSFP) sequence in short-axis and 2-, 3-, and 4-chamber views. The typical imaging parameters used for the cine series are displayed in Table 1. Ten minutes after intravenous administration of 0.075 mmol/kg body weight gadobenate dimeglumine (MultiHance; Bracco), LGE images were acquired using an end-diastolic inversion recovery sequences in short-axis and 2-, 3-, and 4-chamber views.

**Table 1:** Typical imaging parameters employed in this study for cine series.

Acquired voxel size (mm^3^)	1.98 × 1.80 × 6
Reconstructed voxel size (mm^3^)	1.36 × 1.36 × 6
Gap (mm)	4
Slices for full LV coverage	9–10
Echo time (ms)	1.67
Time to repetition (ms)	3.34
Flip angle (°)	60
Sense factor	2.0
Phases per RR interval	25
Temporal resolution (ms)	40

### LVEF analysis

Two investigators (J.E. and J.S.) independently and blindly analyzed each CMR in random order using cvi42 software (Circle Cardiovascular Imaging Inc, Calgary, Alberta, Canada). CMR parameters were indexed to the body surface area (BSA). Evaluation of LVEF was performed in a standard fashion on short-axis cine images at baseline and follow-up^11^.

### LGE analysis

Infarct size was determined in percentage of LGE after drawing endo- and epicardial contours and placing a region of interest into the remote myocardium. The software automatically calculated the mass of myocardial areas with higher signal intensities after choosing a threshold of $\pm$ 2 standard deviations above the remote myocardium. Segmental LGE was qualitatively determined on dedicated short-axis images of the baseline CMR. The presence of LGE was noted with regard to the 17 myocardial segments, as reported by the American Heart Association^12^. Myocardial segments were grouped into segments without LGE (LGE −), segments with LGE (LGE +) and segments with transmural (> 50%) LGE (LGE + +). The presence of microvascular obstruction (MVO), indicated by a no-reflow phenomenon within the infarct zone, was also noted^13,14^.

### Feature tracking analysis

Feature tracking analysis was performed on cine-SSFP images using Segment (Version 2.1.R.6108 from Medviso). As previously described, the software computes interframe deformation fields using endocardial tracking, based on non-rigid image registration^15^. Endocardial contours were manually delineated on the ED images and then automatically tracked throughout the cardiac cycle to determine strain. Left ventricular global and segmental longitudinal strain (LV GLS/ LV SLS) were measured using the 2-, 3- and 4-chamber views. Left ventricular global/segmental circumferential strain (LV GCS/ LV SCS) and global/segmental radial strain (LV GRS/ LV SRS) were determined on three short-axis views (apical, midventricular and basal). The segmental strain analysis was performed once for each patient at BL and FU according to a 17-segment model^12^.

### Statistical analysis

Statistical analysis was conducted using SPSS for Mac (Version 27.0, IBM SPSS Statistics). All measurements were assessed for normality using the Shapiro–Wilk test. Normally distributed data are expressed as mean (± standard deviation) and non-normally distributed data are described using median and interquartile range (IQR). Differences in CMR parameters between patients at baseline and follow-up were determined using a paired-t-test, differences between patients and volunteers at baseline using a t-test for independent variables. Global strain values between patients with and without MVO were also determined using a t-test for independent variables. Wilcoxon-signed-rank-test was used to assess whether strain values between LGE − , LGE + and LGE +  + segments differed between baseline and at six-month follow-up. Receiver operating characteristic (ROC) analysis and Mann–Whitney-U-Test were conducted to portray the ability of strain to predict LGE presence. The statistical analyses performed in this manuscript are hypothesis-generating.

## Results

Strain values could be determined at baseline in all 33 and at six-month follow-up in 32 patients, while one patient was excluded from the analysis due to insufficient image quality at follow-up. No volunteers were excluded from the analysis. Table 2 shows the demographic information of the patients (n = 32) and control group (n = 18). 81.3% of patients and 66.7% of volunteers were male with an average age of 58.9 (± 10.3) and 57.2 (± 7.9) years respectively. The two most common risk factors for coronary artery disease in patients were smoking (53.1%) and hyperlipidemia (37.5%). The most frequently occluded artery in patients was the right coronary artery (RCA) (43.8%). All patients were treated with primary percutaneous coronary intervention.

**Table 2:** Baseline characteristics of the study population.

	STEMI patients (n = 32)	Control group (n = 18)
**Clinical parameters**		
Age, years	58.9 ± 10.3	57.2 ± 7.9
Male sex	26 (81.3)	12 (66.7)
Body surface area, m^2^	2.1 ± 0.2	1.9 ± 0.2
**Infarct characteristics**		
Troponin T, pg/ml	3746 (1225–6144)	
Peak CK, U/L	1671 (877–3626)	
Peak CK-MB, U/L	251 (83–325)	
**Infarct-related artery**		
LAD	13 (40.6)	
CFX	3 (9.4)	
RCA	14 (43.8)	
RCA and CFX	2 (6.2)	
**Cardiovascular risk factors**		
Arterial hypertension	10 (31.3)	
Smoking	17 (53.1)	
Diabetes	4 (12.5)	
Hyperlipidemia	12 (37.5)	
Familiar predisposition	9 (28.1)	
**Medication before infarction**		
ACEI or ARB	11 (34.4)	
Beta-Blocker	12 (37.5)	
Diuretics	3 (9.4)	
Statins	8 (25.0)	
Aspirin/P2Y12-antagonists	10 (31.3)	
**Treatment**		
PCI (%)	32 (100)	
PCI + stent angioplasty	30 (93.8)	
PCI + thrombolysis	2 (6.3)	
**Secondary prevention medication**		
ACEI or ARB	29 (90.6)	
Beta-Blocker	28 (87.5)	
Diuretics	10 (31.3)	
Statins	31 (96.9)	
Aspirin/P2Y12-antagonists	32 (100)	

Numbers are mean ± SD or median (interquartile range) for continuous and n (%) for categorical data. Abbreviations: *STEMI* ST-elevation myocardial infarction, *CK* creatine kinase, *CK-MB* creatine kinase myocardial band, *LAD* left anterior descending coronary artery, *CFX* circumflex coronary artery, *RCA* right coronary artery, *ACEI* angiotensin-converting enzyme inhibitors, *ARB* angiotensin receptor blockers, *PCI* percutaneous coronary intervention.

### CMR parameters after MI and at follow-up

CMR parameters of the patients at baseline and follow-up and of the control group are summarized in Table 3. Compared to the control group, patients showed a reduced LVEF (51.0 ± 11.7 vs. 64.2 ± 7.5%, *p* < 0.001) and stroke volume index (41.3 ± 9.0 vs. 48.4 ± 9.7 ml/m^2^, *p* = 0.012) at baseline, whereas LV mass index (71.5 ± 15.9 vs. 61.1 ± 9.5 g/m^2^, *p* = 0.006) and end-systolic volume index (41.7 ± 16.2 vs. 26.9 ± 7.4 ml/m^2^, *p* < 0.001) were higher. Global strain was within the normal range in the control group and impaired in patients at baseline (LV GLS: −18.6 ± 2.4 vs. −13.8 ± 3.8% (*p* < 0.001), LV GRS: 47.8 ± 8.6 vs. 37.8 ± 11.4 (*p* = 0.002), LV GCS: −18.7 ± 3.8 vs. −14.7 ± 4.0 (*p* = 0.001)). No LGE was detected in the control group, whereas all STEMI patients showed LGE.

**Table 3:** Mean (± SD) CMR parameters of the study population.

	STEMI patients (n = 32)			Control group (n = 18)	
	Baseline	Follow-up	*P* value	Baseline	*P value*
LVEF, %	51.0 ± 11.7	52.7 ± 9.8	0.206	64.2 ± 7.5	< 0.001
LV mass index, g/m^2^	71.5 ± 15.9	64.5 ± 13.3	< 0.001	61.1 ± 9.5	0.006
LVEDVi, ml/m^2^	83.0 ± 17.2	87.3 ± 18.6	0.047	75.4 ± 12.1	0.106
LVSVi, ml/m^2^	41.3 ± 9.0	43.8 ± 5.7	0.101	48.4 ± 9.7	0.012
LVESVi, ml/m^2^	41.7 ± 16.2	43.2 ± 17.1	0.397	26.9 ± 7.4	< 0.001
LV GLS, %	−13.8 ± 3.8	−15.8 ± 4.0	< 0.001	−18.6 ± 2.4	< 0.001
LV GRS, %	37.8 ± 11.4	41.9 ± 12.6	0.006	47.8 ± 8.6	0.002
LV GCS, %	−14.7 ± 4.0	−15.6 ± 3.9	0.023	−18.7 ± 3.8	0.001
Global LGE, %	22.0 ± 8.1	15.6 ± 7.2	< 0.001	/	/

Numbers are mean ± SD for continuous and n (%) for categorical data. Abbreviations: *SD* standard deviation, *LVEF* left ventricular ejection fraction, *LVEDVi* left ventricular end-diastolic volume index, *LVESVi* left ventricular end-systolic volume index, *LVSVi* left ventricular stroke volume index, *LV GLS* left ventricular global longitudinal strain, *LV GRS* left ventricular global radial strain, *LV GCS* left ventricular global circumferential strain.

**Figure 1:**
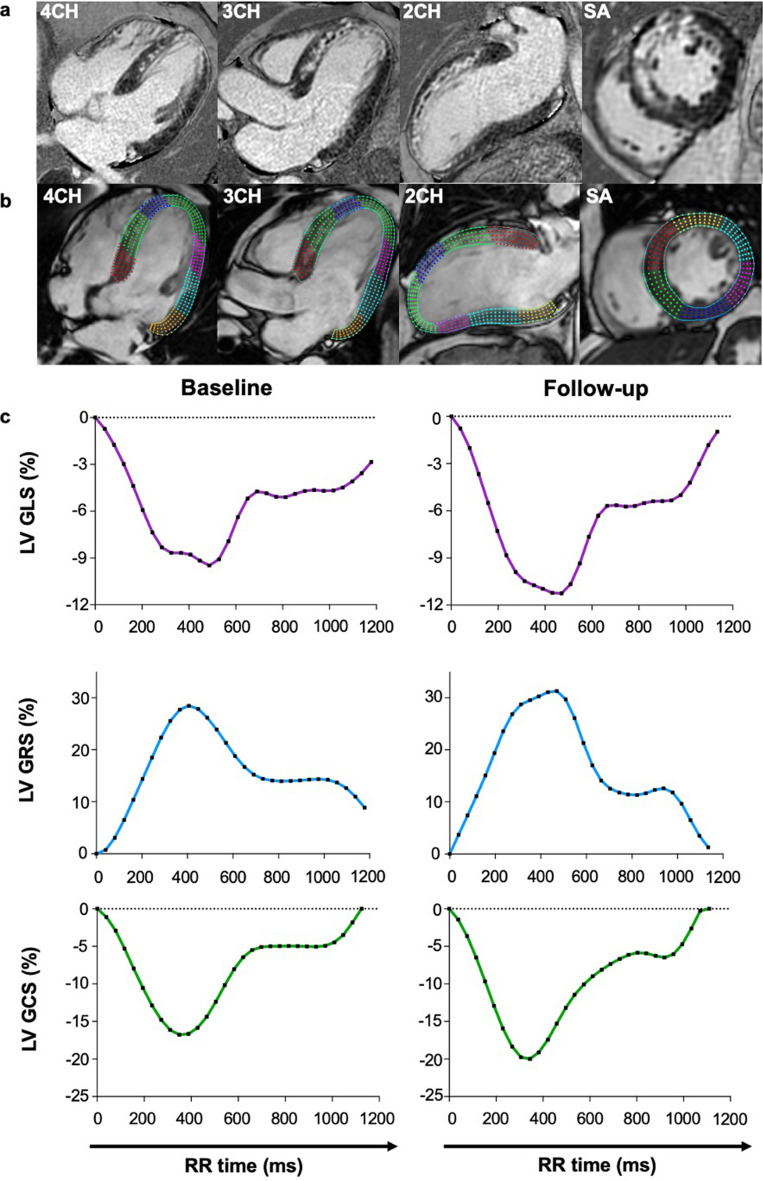
Exemplary LGE-images (**a**), images of the strain analysis (**b**) and strain curves (**c**) of a patient with STEMI in the LAD territory. Abbreviations: *STEMI* ST-elevation myocardial infarction, *LAD* left anterior descending coronary artery, *LV GLS* left ventricular global longitudinal strain, *LV GRS* left ventricular global radial strain, *LV GCS* left ventricular global circumferential strain. Exemplary LGE-images (**a**) and strain curves (**b**) of a patient with STEMI in the LAD territory. In this exemplary study patient, all global strain values were impaired directly after myocardial infarction (LV GLS was −9%, LV GRS was 30% and LV GCS was −17%) and improved at follow-up. LGE showed a subendocardial pattern in the basal anterior wall, a transmural pattern midventricular and a semicircular pattern at the apex. Of the 16 myocardial segments, non-transmural LGE was noted in five and transmural LGE in three segments at baseline.

When comparing patients’ CMR parameters at baseline and follow-up, LVEF (51.0 ± 11.7 vs. 52.7 ± 9.8%, *p* = 0.206) was similar. LV mass index decreased (71.5 ± 15.9 vs. 64.5 ± 13.3 g/m^2^, *p* < 0.001) and LV end-diastolic volume index (83.0 ± 17.2 vs. 87.3 ± 18.6 ml/m^2^, *p* = 0.047) increased. LV stroke volume and LV end-systolic volume indices did not change. Infarct size (%LGE) decreased (22.0 ± 8.1 vs. 15.6 ± 7.2% of LV, *p* < 0.001). All global strain values improved from baseline to six months following MI: LV GLS (−13.8 ± 3.8 vs. −15.8 ± 4.0%, *p* < 0.001); LV GRS from (37.8 ± 11.4 vs. 41.9 ± 12.6%, *p* = 0.006) and LV GCS (−14.7 ± 4.0 vs. −15.6 ± 3.9%, *p* = 0.023). MVO was reported in 16 out of 32 patients (50%). Global strain values differed between patients with and without MVO at baseline and follow-up (GLS: −11.9 ± 3.6 vs. −15.8 ± 3.0% (*p* = 0.002) and −13.8 ± 4.5 vs. −17.8 ± 2.3% (*p* = 0.003)/ GCS: −12.8 ± 4.4% vs. −16.5 ± 2.7% (*p* = 0.008) and −14.0 ± 4.2% vs. −17.1 ± 2.8% (*p* = 0.020)/ GRS: 32.1 ± 11.3% vs. 43.4 ± 8.6% (*p* = 0.003) and 37.0 ± 13.4 vs. 46.8 ± 9.9 (*p* = 0.025), respectively). Images and results of the strain analysis in an exemplary study patient at baseline and follow-up, as well as the correlating LGE images at baseline are illustrated in Fig. 1. This patient suffered from a STEMI due to LAD occlusion. LGE showed a subendocardial pattern in the basal anterior wall, a transmural pattern midventricular and a semicircular pattern at the apex. Moreover, the patient also showed MVO. Of the 17 myocardial segments, non-transmural LGE was noted in five and transmural LGE in three segments at baseline. All global strain values were impaired at baseline: LV GLS was -9%, LV GRS was 30% and LV GCS was −17%. Strain improved from baseline to follow-up, as shown by the strain curves.

### Segmental strain after MI and at six-month follow-up

**Figure 2:**
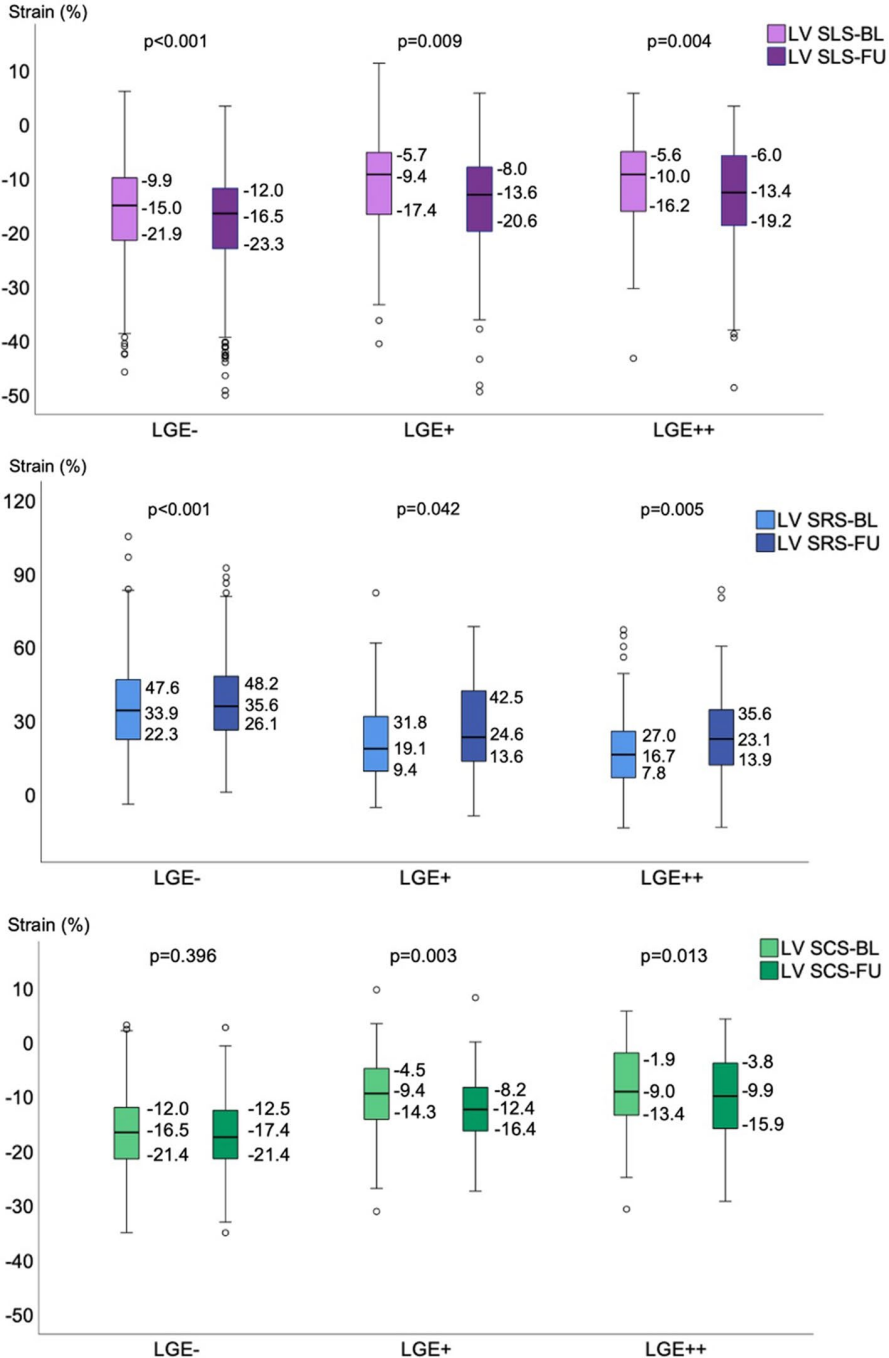
Box plots of the median (interquartile range) segmental strain results of patients at baseline and follow-up, grouped according to the extent of infarction. Abbreviations: *LV SLS* segmental longitudinal strain, *LV SRS* left ventricular segmental radial strain, *LV SCS* left ventricular segmental circumferential strain, *LGE* $-$ myocardial segments without LGE, *LGE* + segments with non-transmural LGE, *LGE* +  + segments with transmural (> 50%) LGE. Box plots of the median (interquartile range) segmental strain results of patients at baseline and follow-up, grouped according to the extent of infarction. LV SLS, LV SRS and LV SCS improved from baseline to six-month follow-up. This improvement was observed in LGE − segments, as well as in LGE + und LGE +  + segments. Only LV SCS in LGE − segments did not change.

Overall, LV SLS improved from a median of −13.5% (interquartile range (IQR): −20.4 to −8.1%) to −15.8% (−22.5 to −10.3%, *p* < 0.001). LV SRS improved from 29.5% (17.6–43.2%) to 33.7% (22.0–46.0%, *p* < 0.001) and LV SCS from −15.1% (−20.4 to −9.3%) to −15.9% (−20.4 to −10.6%, *p* = 0.004). The longitudinal dynamics of segmental strain in patients, grouped according to the extent of LGE, are presented in Fig. 2. LV SLS, LV SRS and LV SCS improved from baseline to six-month follow-up. This improvement was observed in LGE − segments, as well as in LGE + und LGE +  + segments. However, compared to LGE- segments, strain values remained impaired in LGE + / +  + segments. LV SCS showed the largest difference between LGE $-$ and LGE + / +  + segments at baseline and at follow-up (−17.4% (−21.4 to −12.5%) in LGE- segments vs. −11.1% (−16.0 to −5.5%) in LGE + / +  + segments combined, *p* < 0.001). In patients with STEMI of the LCX, baseline LV SCS and LV SRS were markedly impaired in the LCX territory segments (5, 6, 11, 12 and 16^12^) at baseline and improved at follow-up: −10.6 ± 6.1 vs. −16.5 ± 5.6% (*p* = 0.120) and 34.0 ± 19.0 vs. 38.4 ± 12.9% (*p* = 0.750) respectively. There was no relevant change in LV SLS (−16.8 ± 6.9 vs. −16.3 ± 4.0%, *p* = 0.35). In patients with STEMI of the RCA, mean baseline LV SCS and LV SRS were reduced in the RCA territory (segments 3, 4, 9, 10 and 15^12^) and improved from baseline to follow-up: −10.5 ± 3.7 vs. −18.1 ± 3.1% (*p* < 0.001) and 23.3 ± 6.5 vs. 36.3 ± 10.5% (*p* < 0.001), respectively. LV SLS did not significantly improve: −13.5 ± 3.2 vs. −15.8 ± 3.4% (*p* = 0.061). In patients with STEMI of the LAD, mean baseline LV SLS and LV SRS were reduced in LAD territory segments (1, 2, 7, 8, 13 and 14^12^) and improved over time: −10.0 ± 3.7 vs. −16.2 ± 3.9% (*p* < 0.001), 15.1 ± 10.3 vs. 35.0 ± 10.4% (*p* < 0.001), respectively. LV SCS remained impaired (−10.6 ± 6.3 vs. −13.7 ± 3.6%, *p* = 0.136).

### Strain as a predictor of LGE

There was a significant difference in strain at baseline and follow-up between LGE − and LGE + or +  + segments (all *p* < 0.001). However, only LV SCS at follow-up differed significantly between LGE + and LGE +  + segments (−12.4% (−16.4 to −8.2) vs. −9.9% (−15.6 to −3.8), *p* = 0.027), whereas LV SLS, LV SRS and LV SCS at baseline did not.

**Figure 3:**
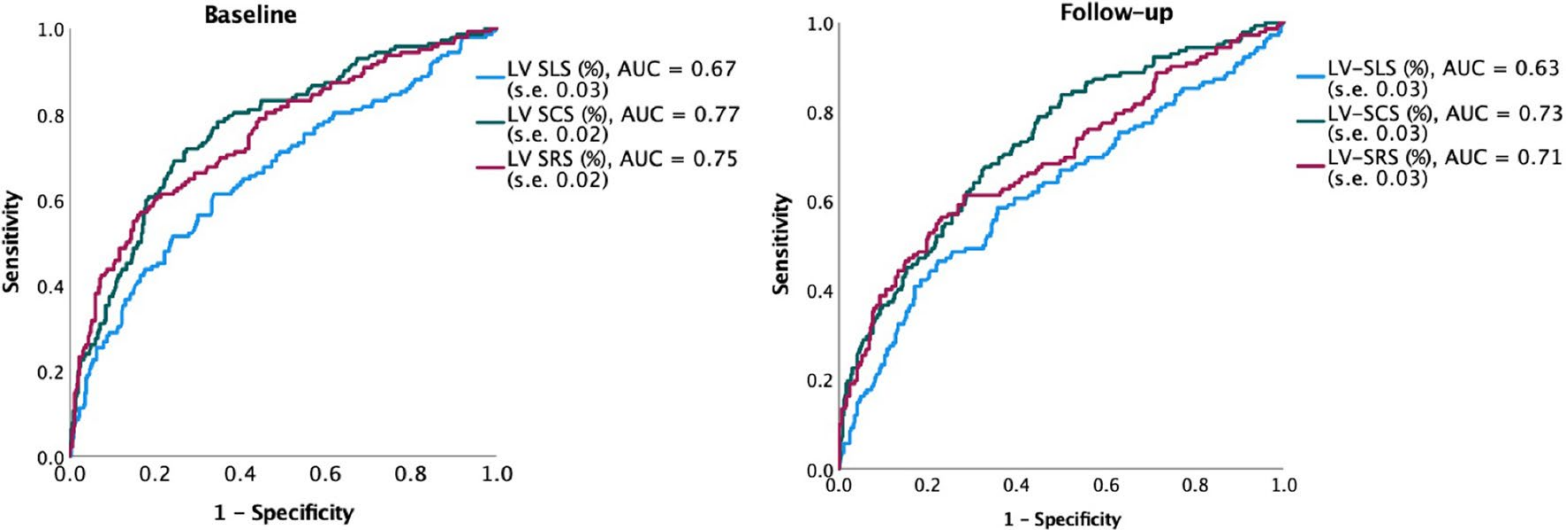
Results of the ROC-analysis on the diagnostic performance of segmental strain to predict LGE at baseline and follow-up. Abbreviations: *LV GLS* left ventricular global longitudinal strain, *LV GRS* left ventricular global radial strain, *LV GCS* left ventricular global circumferential strain, *AUC* area under the curve, s.e.: standard error. Results of the ROC-analysis on the diagnostic performance of segmental strain to predict LGE at baseline and follow-up. These receiver operating curves depict the ability of strain to predict LGE. The highest AUC was observed for LV SCS at baseline and follow-up. The ability of strain to predict LGE decreased by trend from baseline to follow-up.

Figure 3 depicts ROC curves illustrating the diagnostic performance of LV SLS, LV SRS, and LV SCS at baseline and follow-up to distinguish between LGE − and LGE + / +  + segments. LV SLS showed a moderate discriminatory power (area under the curve (AUC): 0.67 (standard error (s.e.) 0.03) at baseline and 0.63 (s.e. 0.03) at follow-up), whereas LV SRS (AUC: 0.75 at baseline (s.e. 0.02) and 0.71 (s.e. 0.03) at follow-up) demonstrated good diagnostic performance. LV SCS performed best in the ROC-analysis and showed the largest AUC of 0.77 (s.e. 0.02) at baseline and 0.73 (s.e. 0.03) at follow-up. Using a cut-off value of −17%, LV SCS at baseline would predict segmental LGE with a sensitivity of 85% and a specificity of 45%, while LV SLS would predict LGE with a sensitivity of 78% and a specificity of 41%. Using a cut-off value of 35%, LV SRS at baseline would predict segmental LGE with a sensitivity of 83% and a specificity of 47%.

## Discussion

CMR is an important modality to evaluate acute and especially chronic MI and enables the precise assessment of the extent of myocardial scar and viability^8^. CMR consistently shows adequate image quality while echocardiography might not always be sufficient for strain analysis, especially in obese patients. Considering potential contraindications of gadolinium-based contrast agent, current research focuses on different parameters to evaluate myocardial viability and recovery after MI without the need for contrast agent administration.

LVEF, the most commonly used parameter to quantify myocardial function, suffers from disadvantages such as the lack of regional functional assessment^3^. Myocardial strain-imaging with FT, however, allows the calculation of regional deformation without the need for acquiring multiple additional sequences. Moreover, it has been proposed that FT CMR could have an incremental prognostic value to predict mortality after MI over LVEF and infarct size^16^ and there is evidence that strain might be able to predict presence of MVO^17^. While global myocardial strain analysis is already implemented in various guidelines, segmental strain analysis is used restrictively due to lower reliability^18^. Our study was designed to provide further insights into the development of global/segmental strain and LVEF from acute to chronic MI and to analyze the ability of strain to predict LGE presence after acute MI and at six-month follow-up.

### Developments in strain and LVEF

In this study, patients with STEMI showed significantly impaired global strain when compared to a healthy control group. However, global and segmental strain improved between the CMR scan following acute MI and at six months. These findings are in agreement with previous reports on global^19^ and segmental strain^20^ development after MI. Interestingly, this improvement was not only seen in myocardial segments with non-transmural LGE, but also in segments with transmural LGE at baseline in this study. A potential reason could be that LGE assessment directly after MI tends to overestimate the extent of non-viable myocardium because of reversible edema and hemorrhage. In this patient group, T1 and T2 relaxation times decreased from acute to chronic infarction as previously published by Tahir et al., indicating that the size of edema/hemorrhage was decreasing^10^. Hence, the size of LGE also decreased during the first weeks after MI, unmasking the underlying myocardial scar and the viable myocardium^21,22^. Therefore, while performing LGE assessment in the acute phase after MI, myocardial segments with reversible edema might have been included and the improvement in strain might reflect the decrease of LGE extent in these segments. The presence of MVO plays an important role in myocardial recovery and prognosis after STEMI besides LGE^14^. In this study cohort, global strain values remained pathological in patients with MVO at BL and FU, signifying that these patients showed prolonged recovery of myocardial function.

In contrast to strain, LVEF of patients did not relevantly change from baseline to follow-up. On the contrary, even though the LVEF was lower in patients than in controls, it remained within the range of “normal values” after MI (51%) and at six-month follow-up (53%)^23^. An explanation could be that the patients included in this study were comparatively young on average (60 years) and did not suffer from any cardiac events before the MI. On the other hand, strain results in this cohort were below the reported range of normal values in healthy volunteers^24^, indicating that strain could be considered as pathologically reduced in contrast to LVEF. This finding is not surprising, as strain is known to be impaired in patients with heart failure or MI while LVEF often remains preserved^6^. Hence, the results of this study support previous observations that strain could allow the detection of subtle changes in myocardial function, not detected by LVEF^6,25^.

### Strain as a predictor of LGE

Segmental strain was significantly impaired in LGE + compared to LGE- segments in patients, at baseline and after six months. Moreover, strain at baseline and follow-up showed good diagnostic accuracy to discriminate between LGE- and LGE + segments in this patient cohort. Using a cut-off value of -17% for LV SLS and SCS and 35% for LV SRS, segmental strain was corresponding to regional LGE in our cohort with high sensitivity (78–85%) and moderate specificity (35–45%).

Only limited knowledge exists on the ability of FT-derived strain to predict LGE at different time points after MI, providing conflicting results. *Khan *et al*.* studied 24 patients around two days after MI and reported lower results on the ability of strain to predict transmural LGE (> 50%) (AUC of 0.736–0.772 for LV SCS and 0.558 to 0.601 for LV SLS)^26^, as seen in this cohort. *Buss *et al*.* also studied patients in the acute phase after MI with an AUC of 0.91 for LV SCS and 0.74 for LV SLS for predicting infarct transmurality $\ge$ 75%^27^. Gräni et al. studied patients approximately 3 days after STEMI with similarly high sensitivity for LV SCS to predict LGE (AUC = 0.84) as found in this cohort^17^. *Yu *et al*.* reported a high accuracy when using strain to predict segments with > 50% LGE (AUC 0.902 for LV SRS, 0.903 for LV SCS and 0.763 for LV SLS) in patients who received CMR one month after MI^28^.

To the best of our knowledge, this is the first study analyzing the longitudinal relationship between strain and LGE from acute to chronic MI. Not surprisingly, the relationship between segmental strain at follow-up and baseline-LGE declined by trend at six months. This development is also influenced by the reduction of LGE extent over time, with LGE overestimating scar in the acute phase of MI^21,22,28^. Hence, strain at follow-up could reflect the improvement of myocardial function in former LGE + segments. This phenomenon could also explain why previous authors, investigating the relationship between strain and different degrees of LGE at different time points after infarction, found conflicting results. Keeping these trends in mind, strain could be considered a valuable quantitative imaging biomarker to follow-up on segmental myocardial function after MI, reflecting an impaired contractility in LGE + segments, but also a possible improvement with decreasing LGE extent.

LV SCS demonstrated the highest accuracy to predict LGE (AUC 0.77 in the acute phase and 0.73 after six months). Previous studies, investigating the relationship between strain and LGE in patients with ischemic and non-ischemic heart disease, similarly reported a stronger relationship between circumferential strain and LGE than between longitudinal strain and LGE^20,28–30^. Longitudinal strain reflects shortening of subendocardial myofibers, whereas circumferential and radial strain reflect the shortening of myofibers located in the epicardium^31,32^. In the early stage of infarction, the subendocardial region suffers from ischemia first due to the remote blood supply^20,33^. Hence, longitudinal strain should be superior to detect subendocardial scar in the early phase of MI, but circumferential strain might better reflect the loss of viability in a transmural infarction due to the scar region reaching the mid-wall. As a result, circumferential strain might detect persistent LGE with higher sensitivity. Further investigations in large patient cohorts are required to confirm if and why the relationship between circumferential strain and LGE could be stronger than for longitudinal or radial strain.

In the current study, segmental strain was consistently impaired in LGE + myocardial segments in patients, showing that strain could be considered a potential substitute for LGE to detect myocardial scar. Nevertheless, in disagreement with previous results^28^, only LV SCS at follow-up differed significantly between myocardial segments with non-transmural (LGE +) and transmural LGE (LGE + +) in this patient cohort, whereas LV SLS and SRS did not. *Yu *et al*.* reported a significant difference in strain results between segments with 0–25%, 25–50% and 50–75% LGE, but not between segments with 50–75% and over 75% LGE. However, they also noted a significant impairment of strain in regions adjacent to the scarred/LGE + segments, with similar values as in the infarcted regions. In a different study, *Stathogiannis *et al*.* concluded that regional strain is reduced in scar areas, but with considerable overlap in strain values between LGE + and LGE- zones^34^. This overlap could have been driven by lower reproducibility of segmental strain results and by overall LV function^34^. We assume that this overlap in strain between scarred regions and adjacent regions might also explain the moderate specificity of strain to detect LGE- segments in our patient cohort. These results also emphasize that segmental strain is not ready to reliably differentiate between transmural and non-transmural LGE. Reproducibility of segmental strain analysis could be a key component that needs to be improved before further assessing strain as a predictor of different LGE patterns and strengths.

## Limitations

This was a single-center study with a small cohort of patients, who completed the baseline and follow-up exams (32 patients). Moreover, the statistical analyses in this manuscript were hypothesis-generating and did not account for multiple testing. Hence, validation is required in confirmatory multi-center studies with larger patient cohorts. The relationship between the quantitative burden of LGE and segmental strain was not investigated, since LGE is often expressed qualitatively in clinical routine and interpreted according to transmurality (> 50% LGE extent)^11^. The focus of this work was on the development of strain after acute MI and not on the development of LGE. We only investigated patients with STEMI, as these patients usually suffer from more severe infarctions with large infarct size, high degree of LGE transmurality and increased chance of MVO^35^. Hence, this patient cohort is more often followed-up after infarction.

## Conclusion

In this study, global and segmental strain was impaired in patients with STEMI compared to healthy controls, but significantly improved between the acute phase of MI and at six-month follow-up while LVEF remained preserved. Patients with MVO showed more attenuated global strain results at baseline and follow-up. Segmental strain improved in LGE- and LGE + segments, but strain was consistently impaired in LGE + segments. The ability of segmental strain to detect LGE was higher during the acute phase of infarction than at six months. Only segmental circumferential strain at follow-up differed significantly between myocardial segments with non-transmural and transmural LGE, while segmental radial and longitudinal strain did not. Especially circumferential strain demonstrated good diagnostic performance for detecting LGE. Segmental strain analysis provides important information on regional damage and recovery after MI and could, with further improvement regarding reproducibility, be a key component to determine regional myocardial function without administration of contrast agent.

## Data Availability

Data supporting the results reported in the manuscript are stored in the Imaging Laboratory of the University Medical Center Hamburg-Eppendorf and are available from the corresponding author on reasonable request.

## Abbreviations

ACEI: Angiotensin-converting enzyme inhibitors
ARB: Angiotensin receptor blockers
CFX: Circumflex coronary artery
CK: Creatine kinase
CK-MB: Creatine kinase myocardial band
CMR: Cardiac magnetic resonance imaging
ED: End-diastole
ES: End-systole
FT: Feature tracking
IQR: Interquartile range
LAD: Left anterior descending coronary artery
LGE: Late gadolinium enhancement
LGE $-$: Myocardial segments without LGE
LGE +: Segments with non-transmural LGE
LGE + +: Segments with transmural (> 50%) LGE.
LVEDVi: Left ventricular end-diastolic volume index
LVEF: Left ventricular ejection fraction
LVESVi: Left ventricular end-systolic volume index
LV GCS: Left ventricular global circumferential strain
LV GLS: Left ventricular global longitudinal strain
LV GRS: Left ventricular global radial strain
LV SCS: Left ventricular segmental circumferential strain
LV SLS: Left ventricular segmental longitudinal strain
LV SRS: Left ventricular segmental radial strain
LVSVi: Left ventricular stroke volume index
MI: Myocardial infarction
MVO: Microvascular obstruction
PCI: Percutaneous coronary intervention
RCA: Right coronary artery
ROC: Receiver operating characteristic
SD: Standard deviation
STEMI: ST-elevation myocardial infarction
S.E.: Standard error
